# Extracellular vesicles and interstitial lung disease in systemic sclerosis: State of the art!

**DOI:** 10.2478/rir-2024-0019

**Published:** 2024-10-21

**Authors:** Jelena Colic, Corrado Campochiaro, Marco Matucci-Cerinic

**Affiliations:** Department of Rheumatology, Institute of Rheumatology, Belgrade, Serbia; Unit of Immunology, Rheumatology, Allergy and Rare Diseases (UNIRAR) & Inflammation, fibrosis and ageing initiative (INFLAGE), IRCCS San Raffaele Hospital, Milano, Italy; Vita Salute San Raffaele University, Milano, Italy

## Introduction

Pulmonary involvement affects more than 70% of systemic sclerosis (SSc) patients, and interstitial lung disease (ILD) is the leading cause of SSc-related disability and mortality The precocious SSc-ILD diagnosis is a challenge since the majority of patients are without symptoms at the early disease stage.^[1]^ Chest computed tomography (CT) is the gold standard for ILD diagnosis, but accessibility to CT in many countries is still difficult, and standardised guidelines for performing CT for the follow-up of ILD progression are still lacking. Moreover, there are no validated clinical markers for diagnosis and prognosis, raising the unmet need for a better understanding of the ILD-SSc pathophysiology.^[2]^

This review aims to identify whether extracellular vesicles (EVs) play a role as biomarkers or pathophysiological players in SSc-ILD.

## Extracellular Vesicles

In the past two decades, the data have shown the EVs biophysical, biochemical and functional heterogeneity. The International Society for Extracellular Vesicles (ISEV) define EV as “particles released from cells, delimited by a lipid bilayer, and without possibility of replication on their own due to the absence of a functional nucleus”. All cells may shed EVs constitutively upon physiological conditions However, EVs may be generated and released upon cellular activation following stimulation with pro-inflammatory cytokines, prothrombotic or proapoptotic signals and exposure to high shear stress.^[3,4]^

Based on their biogenesis, two basic types of EVs have been described: ectosomes and exosomes.^[3]^ The nomenclature of EVs has been changing over time and majority of published articles so far using term “microparticles”(MPs), which are in the literature described as membrane-coated EVs generated from the cells via outward blebbing of the plasma membrane under various conditions (in further text, MPs will be presented as EVs). Even though MPs may be distinguished from other EVs according to the formation mechanism and their content, they are frequently differentiated by size, typically defined as 0.1–1 μm in diameter.^[4]^

EVs are pivotal for intercellular communication, modulating the functions of other cells by delivering intercellular signals through their surface proteins, condensed cargo molecules, and transported lipids and glycans.^[4,5]^ EVs may mediate the communication network between the endothelium, immune cells, and specific organs, including the lungs, acting as effectors of vascular damage and parenchymal fibrosis.^[4]^

To date, EVs originated from different cells (immune cells, platelets, and endothelial cells) and are shown to be associated with inflammatory and autoimmune diseases. The importance of EVs in idiopathic pulmonary fibrosis, sarcoidosis, and hypersensitivity pneumonia has been investigated, but it is still poorly studied in SSc-ILD.^[6]^

## Is There a Link between EVs and SSc-ILD?

**Figure 1:**
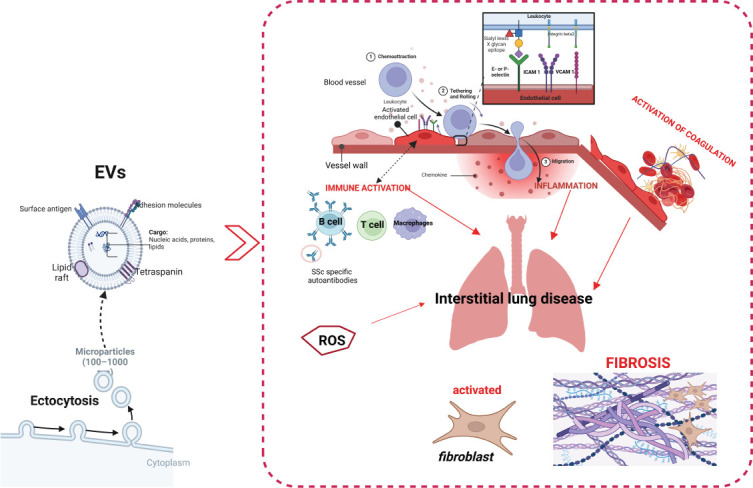
Schematic presentation of the role of Extracellular vesicles in interstitial lung disease development. Figure has been made in Bio render. SSc systemic sclerosis; ICAM 1 Intercellular Adhesion Molecule 1; VCAM1 Vascular cell adhesion protein 1; EVs Extracellular vesicles; ROS reactive oxygen species. Created in BioRender. Colic, J. (2024) BioRender.com/y30l858.

Although the pathophysiology of SSc-ILD still remains elusive, a triad of pathogenic events are considered pivotal-endothelial dysfunction, early inflammation and excessive accumulation of extracellular matrix components (ECM) produced by myofibroblast ^[2]^— where EVs may have a critical role (Figure 1). EVs derived from endothelial cells (EEVs) are considered reliable biomarkers of vascular injury: EVs may initiate and foster endothelial dysfunction, directly disrupting endothelial nitric oxide (NO) production and NO bioavailability, consequently influencing vascular tone.^[7]^ Moreover, transferring chemokine (CXC/CC) receptors and arachidonic acid among cells, EVs may up regulate endothelial injury markers (adhesion molecules, including intracellular adhesion molecule-1 (ICAM-1) and vascular adhesion molecule-1 (VCAM-1) promoting inflammation. Specifically, EEVs promote altered ICAM-1 messenger ribonucleic acid expression after endothelial cells (ECs) interaction followed by enhancement of ICAM1 soluble secretion which in turn fosters interleukin 6 (IL-6) production. EEVs expressing adhesion molecules promote the adhesion of monocytes to ECs in vitro amplifying proinflammatory signaling.^[4,8]^ By carrying the mediators of innate immunity, such as highly pro-inflammatory form –-monomeric C-reactive protein (CRP), EVs release CXC ligand 8 from monocytes, contributing to inflammation dissemination. ^[9]^ Acute phase reactants, specifically IL-6, are critical in the pathogenesis and progression of SSc-ILD.^[2]^ New data support that EVs, upon inflammatory conditions, may transfer interleukin 6 (IL6) to target cells and initiate IL6 soluble secretion from the original cells, while the positive association between EEVs vesiculation and IL6 was observed, supporting a close relation between endothelial injury and proinflammatory and profibrotic pathways mediated by EVs.^[10]^ A growing body of evidence supports EVs as a key player of innate and adaptive immunity, influencing T and B cell development, antigen presentation to lymphocytes, and the immune synapses formed by lymphocytes, subsequently mediating myofibroblast activation and fibrosis.^[2,5]^ The novelty is that EVs, acting as a functional unit with encapsulated bioactive cargo molecules, activate an inflammatory immune response, contributing to SSc-ILD genesis.^[11]^ Oxidative stress, which contributes to ILD genesis, triggers the production of procoagulant EVs by alveolar epithelial cells in vitro. Altered levels of EVs expressing tissue factor (TF) have been found in SSc-ILD. Impaired hemostasis, favouring hypercoagulation, is one of a pivotal event in ILD development. All EVs per se expose phosphatide serine (PS), as a consequence of membrane flipping over apoptosis, which promotes blood clotting. EVs promote and maintain a procoagulant state by the ability to express TF and von Willebrand factor multimers and transfer TF between EVs and other cells.^[4]^

Various growth factors closely implicating in myofibroblast activation, endothelial to mesenchymal transformation (EndoMT) or epithelial to mesenchymal transformation (EMT), which are crucial for ILD SSc genesis, like platelets derived growth factor (PDGF), vascular endothelial growth factor (VEGF) and transforming growth factor β (TGFβ), have been found on the EVs surface or as their internal storage pool.^[2,12]^

The direct profibrotic role of EVs, primarily generated from SSc-ILD patients, has been demonstrated through upregulation of type 1 and 2 collagen expression and activation of the immune system by increasing CC ligand 2 expression, generating human fibroblast fibrotic features.^[13]^ In vivo and animal model studies have shown a significantly increased number of EV originating from SSc lung fibroblasts with the ability to induce fibrotic phenotype in primary lung fibroblasts. Upon master fibrotic stimulus of SSc ILD, EVs cargo was enriched with fibrotic proteins and TGFβ contributing to the ECM development, suggesting EV’s role in the complex signalling of SSc lung fibrosis propagation. Furthermore, suppressing EV shedding (chemically or genetically) has led to diminished lung fibrosis and its severity in SSc murine model, opening a venue for a potential new therapeutic strategy to improve fibrosis.^[14]^

## EVs and Lung Immersion in SSc: Clinical Overview

Clinical studies concerning the associations between circulating EVs and the presence of SSc-ILD are limited and conflicting (Table 1). Studies with greater cohorts of lung affection, demonstrated higher levels of EVs in patients with SSc ILD, specifically shedding light on EEVs in the ILD pathogenesis.^[13,15,16]^ Further on, platelets EVs (PEVs) and monocyte-derived EVs were found to be related to interstitial pneumonia. Only one study having SSc-ILD as a primary endpoint so far has demonstrated that apart from EEVs, PEVs and leucocyte EVs (LEVs), EVs expressing either ICAM-1/TF were linked not only with the presence of ILD but also to the progression of lung disease. The same authors suggested that EVs bearing ICAM1 could be a novel biomarker of ILD progression.^[16]^ Pulmonary functional tests, mainly force vital capacity (FVC%) and diffuse capacity of the lungs for carbon monoxide %, were used for assessing ILD severity, giving divergent results concerning the impact of EVs (Table 1). Three studies have found a significant inverse relation between the total number of EVs, EEVs, LEVs or EVs expressing ICAM-1 with mainly FVC% (Table 1), suggesting their potential contribution to the extent of ILD and mortality.^[2]^

**Table 1:** Clinical association of circulating EVs with the presence of SSc ILD and severity.

EVs origin	EVs labeling	Cohort size	Disease Duration, Years, Mean ± SD	ATA+ *N* (%)	ILD+ (CT) *N* (%)	ILD+ (Xray) *N* (%)	EVslung affection	EVs-PFTs	Refer-ences
Total EVs									
		*N* = 37	13 ± 10	13 (35.1)	18 (46)	NA	-	-	[18]
PEVs	CD42^+^								
ErEVs	CD235^+^								
NEVs	CD66b^+^								
MEVs	CD14^+^								
TcEVs	CD3^+^								
EEVs	CD144^+^								
BcEVs	CD19^+^								
Total EVs							+	+	
EEVs	CD31^+^, CD235^−^, CD41^−^	*N* = 96	7.77 ± 6.53	20 (21.9)	33 (36.3)	NA	+	+	[13]
PEVs	CD41^+^, CD235^−^						-	-	
MEVs	CD14^+^ Annexin V^+^	*N* =4 2	NA	NA	NA	interstitial	MPs-IP+	NA	[19]
PEVs	CD42a^+^					pneumonia	MPs-IP +		
EEVs	CD146^+^	*N* = 121	12 ± 9	15 (12.4)	NA	21 (17.4)	+	+	[15]
LEVs	CD45^+^						-	+	
PEVs	CD42a^+^						-	-	
EEVs	CD105^+^	*N* = 70	6.4 ± 4.0	13 (18.6)	38 (54.3)	NA	-	NA	[20]
MEVs	CD14^+^						-		
PEVs	CD42^+^/CD31						-		
EEVs	CD144^+^	*N* = 59 SSc-ILD *N* = 32	ILD+ 3 (0-29)	26 ILD (19.7%)	32	NA	+	-	[16]
PEVs	CD42b^+^						+	-	
LEVs	CD45^+^						+	-	
EVs-TF	CD142^+^						+	-	
EVs-ICAM1	CD54^+^						+	+	
EVs-HMGB1	HMGB1						+	-	
EEVs	CD144^+^/146+AnnV–						-		
	CD144^+^/146+AnnV+	*N* = 54		NA	ILD (30) 10	NA	-	NA	[7]

SSc, systemic sclerosis; ILD, interstitial lung disease; EVs, extracellular vesicles; PFT, pulmonary functional test; CT, computed tomography; ATA, anti-topoisomerase antibodies; EEVs, endothelial EVs; PEVs, platelets EVs; MEVs, monocytes EVs; NEVs, neutrophile derived EVs; LEVs, leucocyte EVs; ErEVs, erythrocytes EVs; TcEVs, T cells derived EVs; BcEVs, B cells derived EVs; TF, tissue factor; HMGB1, high mobility group box 1; NA, not applicable; + significant association; - without significant association.

The study results should be interpreted cautiously due to the heterogeneity of cohorts, ILD detection methods, labelling of EVs surface marker, EVs separation methodology and a lack of validated and standardized methods. Even though ISEV releases periodically updated specific guidelines of EVs nomenclature, sample processing, separation, characterisation, and functional characterization, as previously reported, none of the analyzed studies didn’t fully follow the minimum information that should be included in scientific papers mostly regarding the separation process.^[3,17]^

Of note, any of analyzed studies in this review haven’t explored relation between EVs and severity of ILD assessed by CT, neither with the type of ILD (nonspecific or usual interstitial pneumonia), nor EVs cargo material with SSc-ILD, thus designing bigger longitudinal studies with well-defined objectives following ISEV statements are needed to overcome limitations.

## Conclusion

Although knowledge about the role of EVs has recently advanced considerably, this research area still presents many challenges, mainly due to lack of validated methodology. Accumulating evidence support the implication of EVs in the complex pathogenesis of lung fibrosis development, mediating endothelial and alveolar injury, inflammation, activation of the hypercoagulable state, and generation of oxidative stress. The direct profibrotic role of EVs in SSc-ILD genesis has been confirmed in vitro so far. Although data from clinical studies are missing and conflicting, EEVs and EVs expressing ICAM1 were found significantly associated with SSc-ILD. However more studies are needed to provide novel insights of EVs as a reliable biomarkers of ILD and its progression, paving the way to new therapeutic avenues.
